# Ferulic acid inhibits LPS-induced apoptosis in bovine mammary epithelial cells by regulating the NF-κB and Nrf2 signalling pathways to restore mitochondrial dynamics and ROS generation

**DOI:** 10.1186/s13567-021-00973-3

**Published:** 2021-07-13

**Authors:** Mingjiang Liu, Chi Zhang, Xiaolong Xu, Xin Zhao, Ziyi Han, Dandan Liu, Ruonan Bo, Jingui Li, Zongping Liu

**Affiliations:** 1 College of Veterinary Medicine, Yangzhou University, Yangzhou, 225009 China; 2 Jiangsu Co-Innovation Center for Prevention and Control of Important Animal Infectious Diseases and Zoonoses, Yangzhou, 225009 China; 3 Beijing Hospital of Traditional Chinese Medicine, Capital Medical University, Beijing, 100010 China

**Keywords:** BMEC, LPS, inflammation, oxidative stress, FA, NF-κB and Nrf2 signals

## Abstract

In bovine mammary epithelial cells (BMECs), a cascade of inflammatory reactions induced by lipopolysaccharide (LPS) has been shown to result in cell injury and apoptosis. The present study aims to reveal the protective effect of ferulic acid (FA) on LPS-induced BMEC apoptosis and explore its potential molecular mechanisms. First, we showed that FA had low cytotoxicity to BMECs and significantly decreased cell apoptosis and the proinflammatory response induced by LPS. Next, FA blocked LPS-induced oxidative stress by restoring the balance of the redox state and inhibiting mitochondrial dysfunction, the main contributor to LPS-induced apoptosis and ROS generation. Furthermore, the relief of inflammation and redox disturbance in the FA preconditioning group were accompanied by weaker NF-κB activation, enhanced Nrf2 activation and maintained cell viability compared to the LPS group. When BMECs were treated with FA alone, we observed that Nrf2 activation was induced before the inhibition of NF-κB activation and that the Keap1–Nrf2 relationship was disturbed. We concluded that FA prevented LPS-induced BMEC apoptosis by reversing the dominant relationship between NF-κB and Nrf2.

## Introduction

During bovine mastitis caused by gram-negative bacteria, the infected mammary glands are always characterized by severe clinical symptoms such as swelling, heat, pain and lactation disorder [1, 2]. The excessive inflammation induced by pathogens and pathogenic components is considered a factor contributing to the destruction of bovine mammary epithelial cells (BMECs), the first line of protection against invading pathogens, the tight junctions of which are the most important structures of the blood-milk barrier [3–5]. Antibiotic treatment is still an indispensable method to combat microbial infections and maintain bovine udder health. However, when uncontrolled by antibiotics, mastitis-causing bacterial virulence factors potentially lead to a sustained inflammatory response and provide evolutionary pressure for the development of antibiotic resistance [6]. Indeed, no effective method has been developed to tackle the antibiotic resistance crisis rather than phasing out the use of antibiotics or promoting their rational use, prompting research on strategies targeting microbial virulence rather than survival [7].

The growing demands for innovative antimicrobials have prompted researchers to develop new methods to combat pathogenic microorganisms. Herbal extracts and antimicrobial peptides have attracted attention due to their immunomodulatory, antibiofilm, anticancer and antimicrobial activities [6, 8, 9]. Based on accumulating evidence, phenolic compounds exhibit remarkable bioactivities, namely, anti-inflammatory, antioxidant, anticancer, and antibacterial activities [10, 11]. Ferulic acid (FA) is a phenolic compound that has been widely used in the pharmaceutical, food, and cosmetics industries due to its low toxicity and abundant physiological functions [12]. To date, no report on the application of FA in bovine mastitis has been published.

Previous studies have established crosstalk between the nuclear transcription factor-kappa B (NF-κB) and NF-E2 p45-related factor 2 (Nrf2) signalling pathways, which regulate cellular responses to oxidative stress and inflammation, respectively [13]. As shown in Figure 6, NF-κB and Nrf2 are both rapid response factors that are maintained in the ‘‘resting’’ state in normal cells but are activated under certain conditions. Under normal conditions, IκB sequesters NF-κB in the cytoplasm, whereas stimuli trigger the degradation of the IκB protein and the subsequent release of NF-κB dimers (the P50:P65 dimer is the primary NF-κB isoform induced by proinflammatory stimuli), which translocate to the nucleus where they bind to specific DNA sequences and promote the transcription of proinflammatory genes [14]. Nrf2 is a low-abundance protein whose half-life (approximately 15–40 min) is controlled by Keap1 via Nrf2 ubiquitination and degradation [15, 16]. Electrophilic reagents or oxidative stress cause the uncoupling of Nrf2 and Keap1, resulting in an accumulation of Nrf2 in the cytoplasm [17, 18]. The accumulated Nrf2 then transfers to the nucleus and interacts with antioxidant response elements (AREs) to address inflammation and oxidant stress [19, 20]. In addition, Nrf2 and NF-κB competitively bind to the transcriptional coactivator cAMP-response-element-binding protein-binding protein (CBP) in the nucleus, playing opposite roles in regulating the expression of inflammatory genes [21, 22]. The interplay between Nrf2 and NF-κB influences cellular responses to inflammation, oxidative stress and immunity, maintaining the stability of cell metabolism and the internal environment [15, 23, 24].

Lipopolysaccharide (LPS) causes an acute and strong inflammatory response in BMECs, which eventually leads to cell damage and apoptosis [25–27]. In this study, we confirmed the protective effect of FA on LPS-induced BMEC apoptosis and elucidated the regulatory mechanism by which FA modulates the activation of NF-κB and Nrf2, the two major factors coordinately controlling cellular inflammation and oxidative stress.

## Materials and methods

### BMEC isolation, cell culture and treatment

BMECs were isolated from lactating cows whose four quarters were free from pathogens, and somatic cell counts were less than 150 000 cells/mL milk for all 4 quarters. The method used for isolating and culturing BMECs was as described in a previous study [28].

Upon reaching 90% confluence, cells were washed twice with phosphate-buffered saline (PBS) and then stimulated with LPS (50 μg/mL) for the indicated times after an incubation with FA (0, 5, 10, or 15 μg/mL) for 2 h. Next, cells were washed three times with PBS and collected for subsequent experiments. FA (National Institutes for Food and Drug Control, > 98% purity, Beijing, China) and LPS (*E. coli* serotype O55:B5, Sigma-Aldrich, St. Louis, MO, USA) were diluted in DMEM/F12 to a final concentration of 1 mg/mL before being added to culture medium to achieve the final concentration required in the respective assays.

### Cell viability assay

Cell viability was measured using the cell counting kit-8 (CCK-8) assay. Briefly, 2 × 10^3^ BMECs/well were seeded into 96-well plates, cultured for 24 h, and then incubated with gradient concentrations of FA (0, 1, 2, 5, 10, 15, 30 or 60 μg/mL) for 24 h. After incubation, 20 μL of the CCK-8 solution (Dojindo Laboratories, Japan) were added to each well of the cell culture plate and incubated for an additional 4 h. Finally, the absorbance was measured at a wavelength of 490 nm with a microplate reader (BioTek, Winooski, VT, USA).

### Flow cytometry analysis

After treatment according to the test requirements, intracellular reactive oxygen species (ROS) production was detected using 2′,7′-dichlorofluorescin diacetate (DCFH-DA, Sigma-Aldrich, St. Louis, MO, USA) probe. Cell apoptosis was assessed using a FITC Annexin V Apoptosis Detection Kit I (BD Biosciences, San Diego, CA, USA) according to the manufacturer’s instructions. The mitochondrial membrane potential (MMP) was measured using the mitochondrial potential sensor 5,5′,6,6′-tetra-chloro-1,1′,3,3′-tetra-ethyl-benz-imidazolyl-carbocyanine iodide (JC-1, Beyotime, Shanghai, China). Cells incubated with the corresponding dyes were then collected for flow cytometry analysis (Beckman Coulter, CA, USA).

### RNA extraction and reverse transcription-polymerase chain reaction

Cells were stimulated with LPS (50 μg/mL) for 12 h after an incubation with the indicated concentrations (0, 5, 10, or 15 μg/mL) of FA in serum-free medium for 2 h. Total RNA was extracted using TRIzol reagent (Invitrogen, Carlsbad, CA, USA), and the concentration and purity of RNA were measured with a spectrophotometer (Thermo Fisher Scientific, Waltham, MA, USA). Total RNA (2 μg) was reverse transcribed to cDNAs according to the manufacturer’s instructions (Vazyme Biotech, Nanjing, China). Quantitative reverse transcriptase PCR (RT-qPCR) was performed with SYBR Green PCR kits (Vazyme Biotech). Briefly, 10 μL of SYBR-Green I master mix, 1 μL of cDNA, 1 μL of primers and 8 μL of RNase-free water were mixed together for reaction and detection. The RT-qPCR thermal cycling protocol was programmed in the CFX96™ Real-Time PCR Detection system (Bio-Rad, Hercules, CA, USA), and the conditions were as follows: an initial denaturation step at 95 °C for 30 s, followed by 40 cycles of denaturation for 5 s at 95 °C and annealing and extension for 30 s at 60 °C.

### Western blot analysis

After treatment according to the test requirements, the total proteins and nucleoproteins were extracted using a total protein extraction kit (Beyotime) and nuclear protein extraction kit (Beyotime), respectively. The protein concentration was quantified using a BCA protein assay kit (Beyotime). Western blot analysis was performed using equal quantities (10–20 μg) of cell extracts diluted in sample buffer. Proteins were separated on 12% gradient SDS-PAGE gels and transferred to PVDF membranes (Merck Millipore, Bedford, MA, USA) that were then hybridized with specific antibodies. The following primary antibodies were purchased from Cell Signaling Technology, Danvers, MA, USA: β-actin (#4970) and histone H3 (#9717). The following primary antibodies were purchased from Abcam, Cambridge, UK: Bcl-2 (ab183656), Bax (ab32503), Kelch-like ECH-associated protein (Keap1, ab196346), P65 (ab16502), p-P65 (ab6503), inhibitor protein α of κB (IκBα, ab32518), p-IκBα (ab133462) and Nrf2 (ab62352). The signals for each protein band were detected using a Chemidoc XRS instrument (Bio-Rad, Marnes-la-Coquette, France). Blots were normalized to β-actin to correct the differences in protein loading, except nucleoproteins and p-IκBα, which were normalized to histone H3 and IκBα, respectively. Densitometry quantification of immunoblot signals was performed using ImageJ software (National Institutes of Health, Bethesda, MD, USA).

### Redox analysis

Cells were stimulated with LPS (50 μg/mL) for 12 h after an incubation with the indicated concentrations (0, 5, 10, or 15 μg/mL) of FA diluted in serum-free medium for 2 h. Then, cells were collected for total protein extraction using commercial colorimetric assay kits (Beyotime) according to the manufacturer’s instructions. The collected proteins were stored at −80 °C until subsequent analysis. Superoxide dismutase (SOD) and glutathione peroxidase (GSH-Px) activities and malondialdehyde (MDA) concentrations were measured using commercial colorimetric assay kits (Jiancheng, Nanjing, China) according to the manufacturer’s instructions. The absorbance was measured at the appropriate wavelengths using a spectrophotometer (BioTek, Winooski, VT, USA).

### Statistical analysis

Unless indicated otherwise, all data were obtained from at least three independent experiments performed in triplicate, and all data were analysed using SPSS 20.0 statistical software. Statistical analyses of the data were carried out using one-way ANOVA followed by Duncan’s test for multiple comparisons and presented as the means and standard errors of the means (SEM). For all analyses, *P* values < 0.05 were considered statistically significant.

## Results

### FA had no cytotoxic effect on BMECs at a concentration of 60 μg/mL

**Figure 1:**
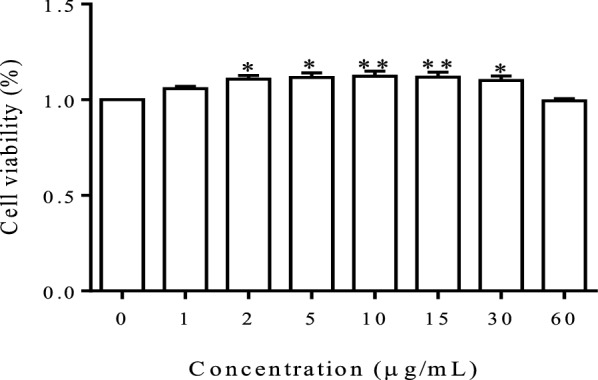
**The effect of FA on BMEC viability.** Cell viability was measured using the CCK-8 assay after an incubation with various concentrations of FA for 24 h. Data are shown as the means ± SEM (*n* = 6), *P < 0.05 compared with the control group and **P < 0.01 compared with the control group.

The CCK-8 assay was used to investigate the cytotoxic effects of FA on BMECs. FA exhibited no cytotoxicity to BMECs at concentrations ranging from 1 to 60 μg/mL. In contrast, it significantly increased cell viability at concentrations ranging from 2 to 30 μg/mL (Figure 1). We used 5, 10, and 15 μg/mL FA in subsequent experiments.

### FA effectively inhibited LPS-induced cell apoptosis and the release of proinflammatory factors

**Figure 2:**
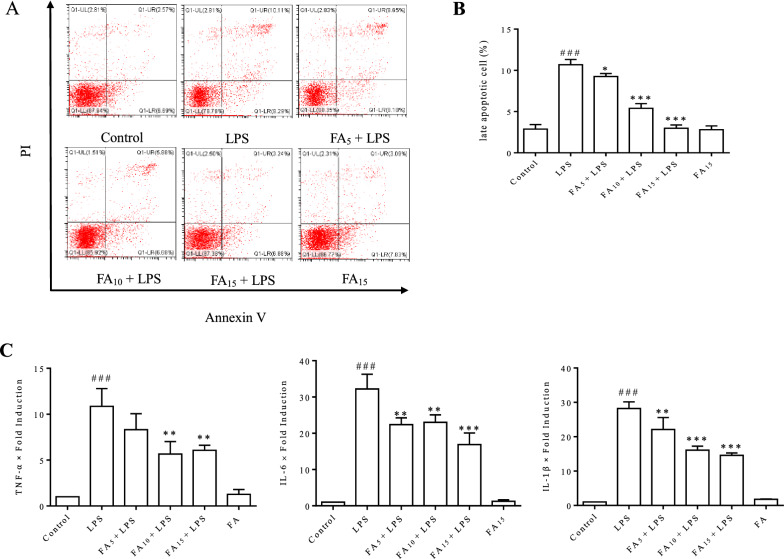
**Effect of FA on LPS-induced BMEC apoptosis and proinflammatory factor release.** Cells were pretreated with the indicated concentrations (0, 5, 10, or 15 μg/mL) of FA diluted with serum-free medium for 2 h before exposure to LPS (50 μg/mL) for 12 h. **A** Cell apoptosis was measured using flow cytometry following annexin V/propidium iodide (PI) staining. Cells in the lower right quadrant (positive for annexin V and negative for PI) were early apoptotic cells. Cells in the upper right (UR) quadrant (both annexin V- and PI-positive) were late apoptotic cells. **B** Late apoptotic cells (cells in the Q1-UR quadrant) were counted. **C** The mRNA expression levels of TNF-α, IL-1β, and IL-6 were quantified using RT-qPCR. The data are presented as means ± SEM (*n* = 3). ^###^*P* < 0.001 compared with the control group, *P < 0.05 compared with the LPS group, and ***P < 0.001 compared with the LPS group.

The cell apoptosis rate and inflammatory cytokine release were examined using flow cytometry and RT-qPCR, respectively, to investigate the protective effect of FA on LPS-induced inflammatory injury in BMECs. As shown in Figure 2A, LPS-induced apoptosis of BMECs was obviously inhibited by FA in a dose-dependent manner. An analysis of late apoptosis rates indicated that 15 μg/mL FA almost completely eliminated cell apoptosis induced by LPS (Figure 2B). Our previous study suggested that the increasing release of inflammatory cytokines plays an important role in LPS-induced apoptosis of BMECs. In the present study, the expression of TNF-α, IL-6, and IL-1β was significantly inhibited by FA, with a more remarkable effect observed in the high-dose group (Figure 2C).

### FA protected BMECs from LPS-induced apoptosis by inhibiting mitochondrial dysfunction and ROS generation

**Figure 3:**
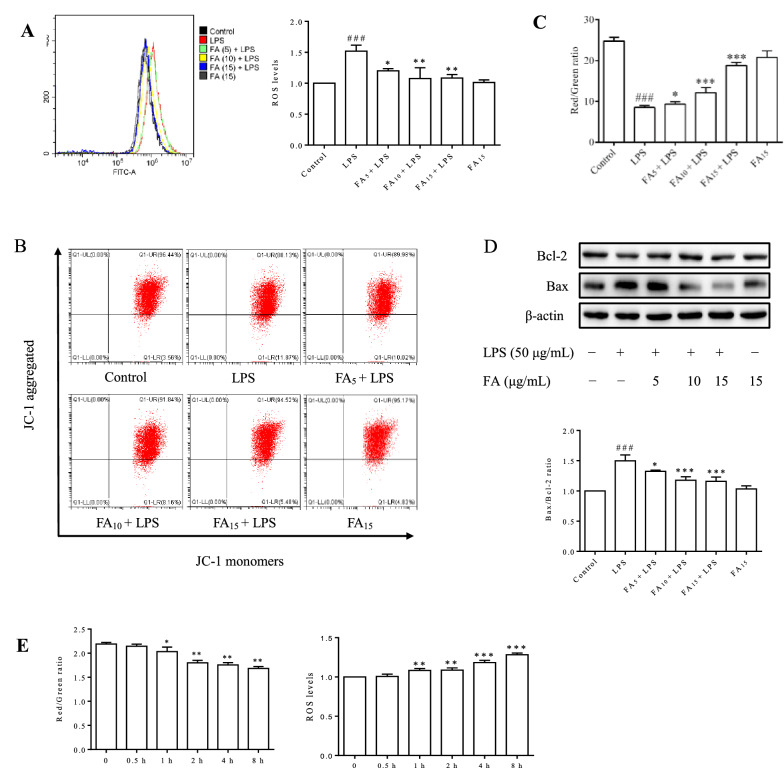
**Effects of FA on LPS-induced BMEC ROS generation and changes in the MMP and Bax/Bcl-2 expression.** Cells were challenged with LPS (50 μg/mL) for 12 h after an incubation with the indicated concentrations (0, 5, 10, or 15 μg/mL) of FA or serum-free media for 2 h (**A**–**D**). **A** The intracellular ROS level was measured using flow cytometry. One plot from three independent experiments is shown, and ROS levels were measured. The data are presented as means ± SEM (*n* = 3). ^###^*P* < 0.001 compared with the control group, *P < 0.05 compared with LPS group, and **P < 0.01 compared with the LPS group. **B** The cellular MMP was determined by performing a bivariate JC-1 analysis using flow cytometry. Signals in the upper right quadrant represent the aggregated red-emitting JC-1 in the matrix of undamaged mitochondria with a normal MMP, while signals in the lower right quadrant represent the green-emitting JC-1 monomers in the cytoplasm of cells with damaged mitochondria. **C** The ratios of red to green fluorescence intensity in different groups are presented, and the values represent the magnitude of the MMP. The data are presented as means ± SEM (*n* = 3). ^###^*P* < 0.001 compared with the control group, *P < 0.05 compared with the LPS group, and ***P < 0.001 compared with the LPS group. **D** The Bax/Bcl-2 expression levels were quantified using Western blot analysis. The data are presented as means ± SEM (*n* = 3). ^###^*P* < 0.001 compared with the control group, *P < 0.05 compared with the LPS group, and ***P < 0.001 compared with the LPS group. **E** The dynamic changes in cellular MMP and intracellular ROS levels were analysed using flow cytometry after stimulation with LPS (50 μg/mL) for different times. The data are presented as means ± SEM (*n* = 3). *P < 0.05 compared with the control group, **P < 0.01 compared with the control group, and *** *P* < 0.001 compared with the control group.

LPS-induced ROS generation and MMP loss were detected using flow cytometry. As shown in Figure 3A, when BMECs were stimulated with LPS, a significant increase in intracellular ROS levels, a critical indicator of oxidative stress, was observed. The cellular MMP was significantly decreased by LPS treatment (*P* < 0.001) (Figures 3B and C). In addition, the decreased Bax/Bcl-2 ratio indicated the initiation of the cell apoptotic process (Figure 3D), consistent with the result presented in Figure 3A. Therefore, we concluded that FA effectively decreased cell apoptosis by preserving the MMP and reducing intracellular ROS generation.

As shown in Figure 3E, the significant increase in intracellular ROS levels (*P* < 0.01) coincided with the decreased MMP (*P* < 0.05) after stimulation with LPS for 1 h. At 0.5 h, no changes were observed in the cellular MMP and ROS generation. We determined that mitochondria were the main source of LPS-induced intracellular ROS production.

### FA protected BMECs from LPS-induced oxidative stress

**Figure 4:**
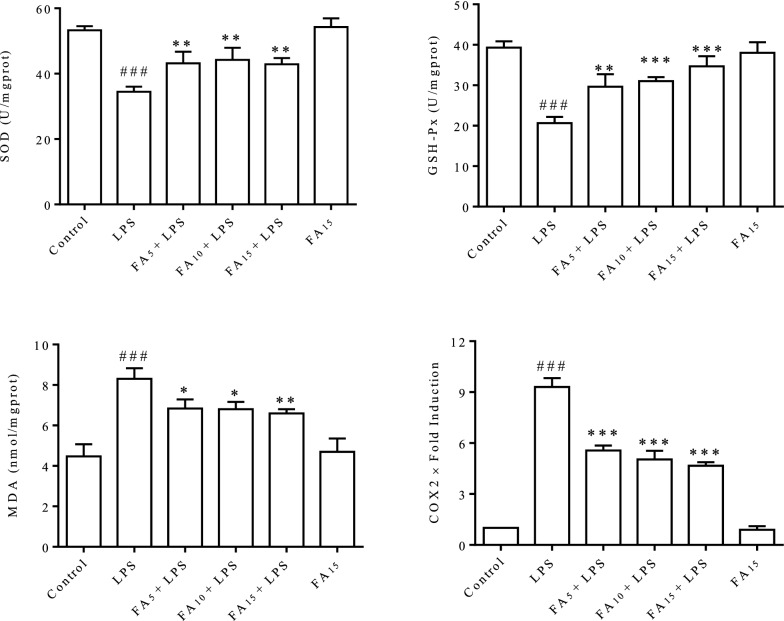
**FA effectively rebalanced the redox state of BMECs that was disturbed by LPS.** Cells were stimulated with LPS (50 μg/mL) for 12 h after a 2 h incubation with the indicated concentrations (0, 5, 10, or 15 μg/mL) of FA diluted in serum-free medium. Cell contents and total RNA were extracted and prepared. The activities of SOD and GSH-Px and the MDA content were detected using commercial kits. COX2 expression was detected using RT-qPCR. The data are presented as means ± SEM (*n* = 3). ^###^*P* < 0.001 compared with the control group, *P < 0.05 compared with the LPS group, **P < 0.01 compared with the LPS group, and ***P < 0.001 compared with the LPS group.

The SOD and GSH-Px activities and MDA content were detected. As shown in Figure 4, the intracellular SOD and GSH-Px activities were significantly reduced, and the MDA content was significantly increased after stimulation with LPS for 12 h, indicating a severe redox imbalance. FA interference significantly rebalanced the redox state by reducing the consumption of SOD and GSH-Px. The expression of cyclooxygenase-2 (COX2), an important factor contributing to inflammation and oxidative stress, was evaluated using RT-qPCR. Significantly increased expression of COX2 was detected in cells stimulated with LPS for 12 h, and this change was significantly inhibited by FA. These effects were dose-dependent, with the higher dose group exhibiting the best effects.

### FA reversed the dominant relationship between NF-κB and Nrf2 induced by LPS

**Figure 5:**
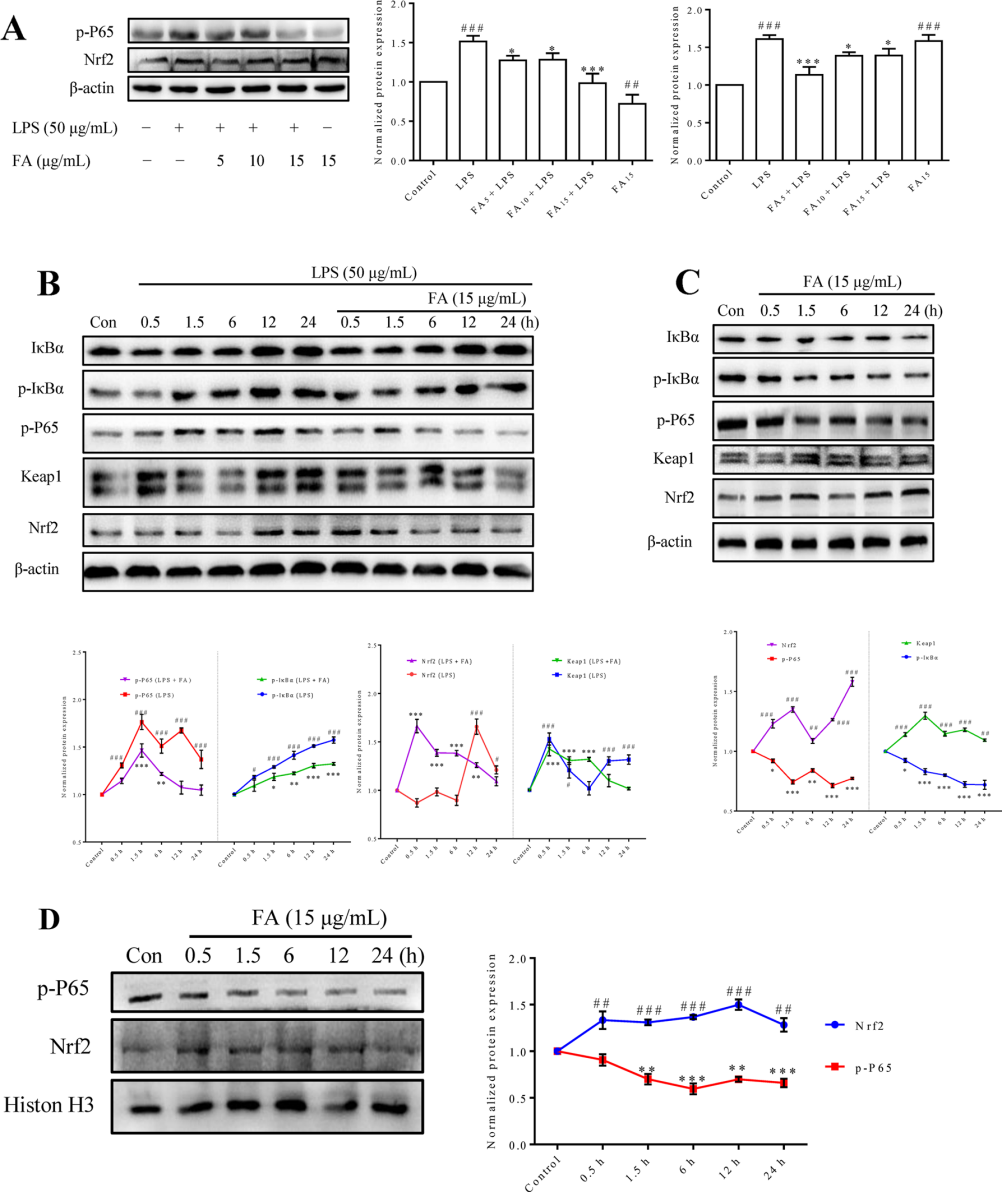
**Effect of FA on the NF-κB and Nrf2 signalling pathways in BMECs cultured in the presence or absence of LPS.** Cells were treated with LPS and/or different doses of FA for the indicated times before total protein (**A**–**C**) or nucleoprotein (**D**) extracts were prepared. Protein expression was quantified using Western blot analysis. **A** Cells were challenged with LPS (50 μg/mL) for 12 h after a 2 h incubation with the indicated concentrations (0, 5, 10, or 15 μg/mL) of FA in serum-free media, and then the levels of p-P65 and Nrf2 was detected. **B** Cells were challenged with LPS (50 μg/mL) for different times after a 2 h incubation with FA (15 μg/mL) or serum-free media, and then the levels of IκBα, p-IκBα, p-P65, Nrf2 and Keap-1 was detected. **C** Cells were incubated with FA (15 μg/mL) for different times, and then the levels of IκBα, p-IκBα, p-p65, Keap1 and Nrf2 were detected. **D** Cells were incubated with FA (15 μg/mL) for different times, and then the levels of the nucleoproteins p-p65 and Nrf2 were detected. One representative Western blot among three independent experiments is shown, and the expression of the studied proteins was normalized to β-actin or Histone H3. Data are shown as the means ± SEM (*n* = 3). Con: control group. ^##^*P* < 0.01, ^###^*P* < 0.001, *P < 0.05, **P < 0.01, and ***P < 0.001 compared with the control group.

The expression of critical proteins, including NF-κB and Nrf2, two major proteins involved in critical signalling pathways, was detected using Western blotting. As shown in Figure 5A, the increased levels of p-P65 and Nrf2 induced by LPS stimulation were significantly decreased by FA. More interestingly, FA significantly reduced p-P65 levels and increased Nrf2 expression, even in normal BMECs. Then, we further investigated the LPS-induced dynamic changes in IκBα, p-IκBα, p-P65, Keap1 and Nrf2 activation at different time points and the regulatory effects of FA. As shown in Figure 5B, LPS induced significant increases in p-IκBα and p-P65 levels at all time points. In groups pretreated with FA, the level of p-P65 was almost completely suppressed, except at 1.5 h and 6 h, and p-IκBα levels remained significantly increased except at 0.5 h, although the increasing tendency appeared to be inhibited. In addition, no obvious change in Nrf2 expression was observed at the early stage of LPS stimulation, while a significant increase was observed after 12 h. After pretreatment with FA, the significant increase in Nrf2 expression persisted for up to 12 h; a rapid increase occurred at 0.5 h and then gradually decreased. In addition, the expression of Keap1 was significantly increased after stimulation with LPS, and a similar trend in Keap1 expression was observed in cells pretreated with or without FA.

Dynamic changes in the levels of IκBα, p-IκBα, p-P65, Keap1 and Nrf2 were detected after cells were incubated with FA for different times to evaluate the potential mechanism by which FA regulates the NF-κB and Nrf2 signalling pathways in normal BMECs. As shown in Figure 5C, the levels of p-IκBα and p-P65 decreased gradually, while the expression of Keap1 and Nrf2 increased significantly at all time points. Then, the endonuclear contents of p-P65 and Nrf2 were detected. As shown in Figure 5D, the nuclear content of Nrf2 was significantly increased at all time points, while the nuclear content of p-P65 was significantly decreased after 0.5 h.

## Discussion

In our previous in vitro study, we determined that the LPS-induced destruction of BMEC tight junctions was mainly due to alterations in the cellular morphological structure and apoptosis [26]. In the present study, FA was not cytotoxic to BMECs at concentrations less than 60 μg/mL (Figure 1), and FA effectively protected against LPS-induced cell apoptosis. FA (15 μg/mL) almost completely eliminated the adverse effect of LPS (Figures 2A and B).

The possible regulatory mechanism of FA in LPS-induced cell apoptosis was explored. The increased generation of proinflammatory cytokines (Figure 2C) and intracellular ROS (Figure 3A) induced by LPS was significantly inhibited by FA in a dose-dependent manner. As an ROS burst can lead to mitochondrial destruction and the ratio of the relative expression of Bax/Bcl-2 plays a central role in the mitochondrion-dependent apoptotic program [29], the MMP and Bax/Bcl-2 ratio were detected in BMECs. The decreased MMP (Figures 3B and C) and increased Bax/Bcl-2 expression ratio (Figure 3D) in LPS-stimulated BMECs indicated that LPS-induced cell apoptosis depended on the mitochondria. The obvious improvement in the levels of oxidative stress markers (SOD, GSH-Px, and MDA) confirmed the antioxidant activity of FA (Figure 4). Our results revealed that FA protected BMECs from apoptosis by inhibiting LPS-induced inflammation and oxidative stress.

ROS are the by-product of normal mitochondrial metabolism and homeostasis [30]. When exposed to microbial and inflammatory stimuli, some types of cells produce excess ROS in the mitochondria for self-defence in a process termed “oxidative burst” that is mediated by the NADPH oxidase complex (NOX), which may lead to cell damage [31, 32]. As shown in Figure 3E, intracellular ROS production and the MMP was unchanged after 0.5 h of stimulation with LPS, whereas both a significant increase in ROS and a loss of MMP concomitantly occurred and were negatively correlated after 1 h, indicating that LPS-induced ROS generation mainly occurred in the mitochondria. In mouse macrophages (RAW264.7), the LPS-induced increase in ROS production appeared at 0.5 h and was attributed to the activation of the NOX complex (data not shown), which produces ROS earlier than mitochondrial dysfunction. Thus, we concluded that the significant increase in ROS levels induced by LPS in BMECs was mainly due to mitochondrial injury rather than NOX complex activation.

The activation of both NF-κB and Nrf2 was enhanced in BMECs after 12 h of LPS treatment. However, the increased activation of Nrf2 did not completely abrogate the proinflammatory effects of NF-κB during this process (Figure 5A). The LPS-induced activation of NF-κB and Nrf2 was significantly inhibited by FA. Interestingly, FA promoted the activation of Nrf2 while inhibiting NF-κB in normal BMECs (Figure 5A). Dynamic changes in the levels of critical proteins were detected to better understand the mechanisms by which FA regulates the LPS-activated NF-κB and Nrf2 signalling pathways. As shown in Figure 5B, LPS-induced NF-κB activation was significantly increased in cells without the FA pretreatment at all time points. Meanwhile, Nrf2 activity was initially inhibited and then activated. Based on these results and the coordinated activity of Nrf2/NF-κB in cell antioxidant and inflammatory responses [15, 33, 34], we hypothesized that Nrf2 activation was unable to offset the cellular inflammation and oxidative stress mediated by LPS-activated NF-κB, because its activation was significantly increased in the late phase. When cells were pretreated with FA, a rapid increase in Nrf2 activity emerged at the initial stage of LPS stimulation and lasted for 12 h, and NF-κB activation was obviously inhibited. Therefore, we presumed that the underlying mechanism by which FA protects BMECs from LPS-induced cell dysfunction and apoptosis is to promote Nrf2 activation and inhibit NF-κB activation.

**Figure 6:**
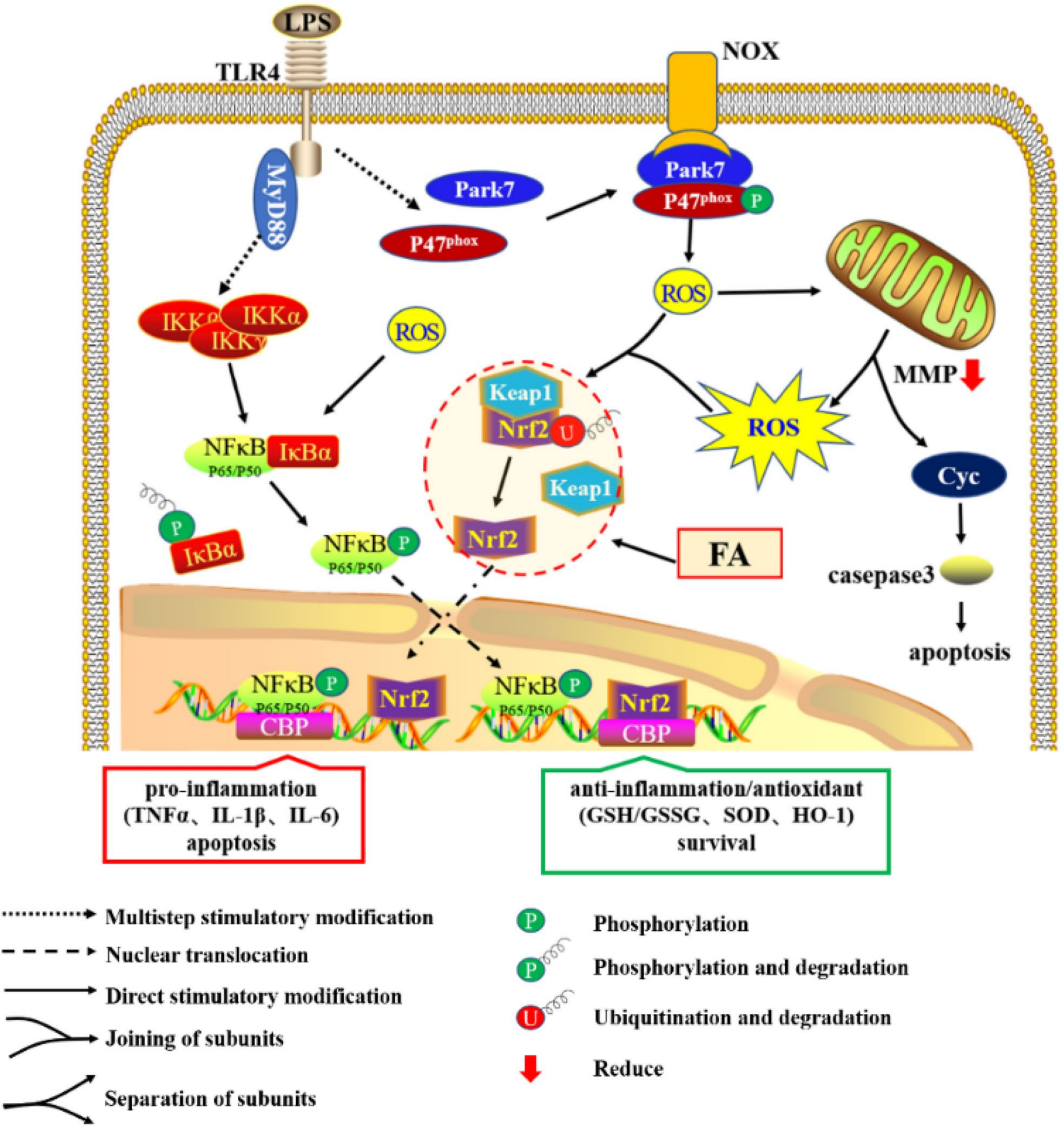
**Effect of FA on LPS-induced cellular inflammation, oxidative stress and apoptosis.** Through the canonical NF-κB pathway, TLR4 recognizes extracellular LPS and transduces signals via MyD88 to activate NF-κB-mediated proinflammatory gene transcription. In addition, ligated TLR4 promotes the interaction between Park7 and P47^phox^, which increases NOX-mediated ROS generation. Subsequently, excessive ROS accumulation in the cytoplasm leads to a loss of the MMP, mitochondria-dependent apoptosis and uncoupling of Nrf2-Keap1 and IκBα-NF-κB. Most notably, although dissociated Nrf2 is responsible for anti-inflammatory and antioxidant activity, its levels were increased after 12 h of LPS treatment but did not block the inflammation and oxidative stress induced by NF-κB in the early stage. However, FA interference advances the activation of Nrf2, consequently relieving the inflammatory response and oxidative stress to inhibit cellular apoptosis and promote BMEC survival.

Then, the function of FA in enhancing Nrf2 activation and suppressing NF-κB activation in normal BMECs was confirmed (Figure 5C). In addition, endonuclear Nrf2 levels were significantly increased after an incubation with FA for 0.5 h, while a decrease in p-P65 levels emerged at 1.5 h (Figure 5D). Therefore, we determined that FA inhibited NF-κB by promoting Nrf2 activation in the early stage, which played a dominant role in resisting LPS-induced damage. Surprisingly, the cumulative effect of Keap1 synchronized with Nrf2 in the early phase (Figure 5C), as these proteins have been reported to antagonize each other [35]. The trend of Keap1 expression was also similar to that observed in the presence of LPS, as shown in Figure 5B. This phenomenon indicated that the Nrf2-Keap1 interaction was disrupted by FA. Thus, we presumed that FA regulates the relationship of NF-κB and Nrf2 signalling by disturbing the Nrf2-Keap1 interaction (Figure 6).

COX2, a prostaglandin-endoperoxidase 2 synthase, plays a central role in various inflammatory processes and ROS generation [36, 37], and it reacts with Keap1 as a strong electrophile to activate Nrf2 and inhibit NF-κB activity [38]. Prostaglandins, which are downstream of COX2, regulate Bax-dependent apoptosis in several cell types and/or contexts [39, 40]. In view of LPS-induced COX2 overexpression (Figure 4) and cell apoptosis in BMECs (Figure 2), the possible mechanisms of COX2 in LPS-induced ROS generation and apoptosis of BMECs require further investigation.

In addition to apoptosis, pyroptosis and necroptosis are additional inflammatory programmed cell death pathways. In contrast to apoptosis, pyroptosis is characterized by swelling, rupture, release of the cellular contents (processed IL-1β and IL-18) and potent proinflammatory effects, which are critical for controlling microbial infection [41–43]. ROS-mediated NOD-like receptor family pyrin domain-containing 3 (NLRP3) inflammasome activation plays a fundamental role in pyroptosis [44–46]. Due to microstructural destruction, significant IL-1β release and ROS generation were observed in BMECs treated with LPS in our study [26]. We speculated that pyroptosis and apoptosis were concomitantly activated in this process, and the mechanisms of pyroptosis in LPS-induced inflammatory injury in BMECs require further study.

In summary, this study revealed that FA prevented LPS-induced apoptosis in BMECs by promoting Nrf2 activation in the initial stage and inhibiting LPS-induced NF-κB activation. During this process, the Keap1-Nrf2 relationship is a vital target of FA. In addition to Keap1, Nrf2 is controlled by a complex transcriptional/epigenetic and posttranslational network [47], and thus the exact mechanism by which FA regulates the crosstalk between NF-κB and Nrf2 requires further investigation. Collectively, this study describes a potential clinical application of FA as a treatment for bovine mastitis.

## Abbreviations

BMEC: bovine mammary epithelial cells
LPS: lipopolysaccharide
FA: ferulic acid
*E. coli*: *Escherichia coli*
NF-κB: nuclear transcription factor-kappa B
Nrf2: NF-E2 p45-related factor 2
ROS: reactive oxygen species
PBS: phosphate-buffered saline
CCK-8: cell counting kit-8
DCFH-DA: 2′,7′-Dichlorofluorescin diacetate
MMP: mitochondrial membrane potential
RT-qPCR: quantitative reverse transcriptase PCR
JC-1: 5,5′,6,6′-Tetra-chloro-1,1′,3,3′-tetra-ethyl-benz-imidazolyl-carbocyanine iodide
SOD: superoxide dismutase
GSH-Px: glutathione peroxidase
MDA: malondialdehyde
SEM: standard error of the mean
COX2: cyclooxygenase-2
Keap1: Kelch-like ECH-associated protein
IκBα: inhibitor protein α of κB
NOX: NADPH oxidase complex
AREs: antioxidant response elements
CBP: cAMP response element binding protein
